# Baseline Anemia and Clinical Outcomes Following Transcatheter Edge-to-Edge Mitral Valve Repair: A Systematic Review and Meta-Analysis

**DOI:** 10.7759/cureus.114910

**Published:** 2026-08-21

**Authors:** Abdulhafiz Hakmi, Ahmad Alhameed, Albatoul Atasi, Obaida Serdar, Wael Al-Ghabra

**Affiliations:** 1 Emergency, Hillingdon Hospitals NHS Foundation Trust, London, GBR; 2 Dentistry, Ajman University, Ajman, ARE; 3 Orthopedics and Traumatology, Hillingdon Hospitals NHS Foundation Trust, London, GBR; 4 Cardiology, The Hillingdon Hospital, London, GBR

**Keywords:** anemia, clinical outcomes, mitra clip, mitral regurgitation (mr), mitral valve transcatheter edge-to-edge repair, mortality, systematic review and meta analysis, transcatheter edge-to-edge repair (teer)

## Abstract

Anemia is common among patients undergoing transcatheter edge-to-edge mitral valve repair (TEER) and may be associated with adverse clinical outcomes. However, no previous systematic review or meta-analysis has specifically evaluated clinical outcomes according to baseline anemia status in this population. We conducted a systematic review and meta-analysis to evaluate the association between baseline anemia and clinical outcomes after mitral TEER. A systematic search of PubMed, Embase, the Cochrane Library, ClinicalTrials.gov, Cumulative Index to Nursing and Allied Health Literature (CINAHL), Virtual Health Library (VHL), and Google Scholar was performed to identify studies comparing outcomes in patients with and without baseline anemia who underwent mitral TEER. Random-effects meta-analyses were performed for outcomes with sufficiently homogeneous data, while outcomes unsuitable for quantitative pooling were narratively synthesized. Study quality was assessed using the Newcastle-Ottawa Scale (NOS). Six observational studies involving 34,812 patients were included. Baseline anemia was associated with significantly increased long-term mortality following TEER in the primary pooled analysis (hazard ratio (HR) 1.86, 95% CI 1.57-2.22), with consistent findings in the sensitivity analysis (HR 1.89, 95% CI 1.63-2.21). Baseline anemia was also associated with higher in-hospital mortality (OR 1.42, 95% CI 1.13-1.77). In contrast, pooled analyses of acute kidney injury, long-term composite outcome, and pericardial complications did not demonstrate statistical significance. Baseline preprocedural anemia is associated with increased mortality following transcatheter edge-to-edge mitral valve repair and may identify patients at higher procedural risk. Further prospective studies are needed to determine whether preprocedural optimization of anemia improves clinical outcomes.

## Introduction and background

Mitral regurgitation (MR) is one of the most common valvular heart diseases, particularly in older adults, affecting nearly 10% of individuals aged 75 years or older with moderate or severe disease [1,2]. Without treatment, symptomatic MR is associated with progressive clinical deterioration and may eventually lead to heart failure and death [3]. A considerable proportion of patients with severe symptomatic MR are not suitable candidates for conventional surgery because of advanced age, impaired ventricular function, or substantial comorbidity burden, highlighting the need for less invasive treatment strategies [4]. This unmet clinical need has led to the increasing development and adoption of transcatheter mitral interventions, particularly TEER. The MitraClip system, the most extensively studied transcatheter edge-to-edge mitral valve repair (TEER) device, is based on the surgical edge-to-edge technique first described by Alfieri and was subsequently supported by preclinical and early clinical studies demonstrating the feasibility of this approach [5,6]. Registry data have subsequently demonstrated increasing adoption of transcatheter mitral valve repair in contemporary practice [7]. TEER is performed percutaneously via femoral venous access and transseptal puncture, with approximation of the mitral leaflets under echocardiographic and fluoroscopic guidance to reduce mitral regurgitation [8].

Patients undergoing TEER are generally older and medically complex, with multiple comorbidities that may influence procedural and longer-term outcomes [8]. Anemia is particularly common among patients undergoing mitral TEER, with an observational cohort reporting a prevalence of 53.9% [9]. Anemia is also common in elderly patients with structural heart disease [10] and has been associated with higher postoperative morbidity and mortality in patients undergoing cardiac surgery [11]. More broadly, anemia has been linked to adverse outcomes in several cardiovascular settings, including heart failure, acute coronary syndrome, percutaneous coronary intervention, and cardiac surgery [12,13]. In patients undergoing TEER, several observational studies have reported that baseline anemia or lower hemoglobin levels are associated with worse clinical outcomes and may represent adverse prognostic markers [14].

Although previous observational studies have evaluated baseline anemia in patients undergoing mitral TEER, no prior systematic review or meta-analysis has synthesized the available evidence comparing clinical outcomes between patients with and without baseline anemia. Moreover, the existing studies differ in follow-up duration, reported endpoints, and analytical methods, limiting the ability to determine the overall prognostic significance of baseline anemia and to draw definitive conclusions from individual reports alone. Therefore, we performed this systematic review and meta-analysis to evaluate the association between baseline preprocedural anemia and clinical outcomes following mitral TEER.

## Review

Methods

We conducted and reported this systematic review and meta-analysis in accordance with the Preferred Reporting Items for Systematic reviews and Meta-Analyses (PRISMA) 2020 statement [15]. The review protocol was not prospectively registered. 

Information sources

We searched PubMed, Ovid Embase, the Cochrane Library, ClinicalTrials.gov, Cumulative Index to Nursing and Allied Health Literature (CINAHL), Virtual Health Library (VHL), and Google Scholar from database inception to 18 March 2026. Google Scholar was additionally searched using predefined search queries, with the first six pages of results screened for each query. Reference lists of included studies and relevant reviews were also screened to identify additional eligible reports.

Search strategy

The complete database-specific search strategies and numbers of records retrieved are presented in Table 1, with the stepwise Ovid Embase search strategy provided in Table 2.

**Table 1:** Full database search strategies and records retrieved. Scopus and Web of Science were not searched because institutional access was unavailable. System for Information on Grey Literature in Europe (SIGLE) was not searched because grey literature was excluded according to the predefined eligibility criteria. CINAHL: Cumulative Index to Nursing and Allied Health Literature; VHL: Virtual Health Library.

Database	Search strategy	Records retrieved/notes
PubMed	(("Mitral Valve Insufficiency"[Mesh] OR mitral regurgitation[tiab] OR "mitral valve insufficiency"[tiab] OR "mitral incompetence"[tiab] OR "mitral valve incompetence"[tiab] OR "mitral insufficiency"[tiab]) AND ("Anemia"[Mesh] OR anemia[tiab] OR anaemia[tiab] OR hemoglobin[tiab] OR haemoglobin[tiab] OR "low hemoglobin"[tiab] OR "low haemoglobin"[tiab]) AND (TEER[tiab] OR "transcatheter edge-to-edge repair"[tiab] OR "transcatheter mitral edge-to-edge repair"[tiab] OR "edge-to-edge repair"[tiab] OR MitraClip[tiab] OR PASCAL[tiab] OR "transcatheter mitral valve repair"[tiab] OR "percutaneous mitral valve repair"[tiab]))	39
CINAHL	("mitral regurgitation" OR "mitral valve insufficiency" OR "mitral incompetence" OR "mitral valve incompetence" OR "mitral insufficiency") AND (anemia OR anaemia OR hemoglobin OR haemoglobin OR "low hemoglobin" OR "low haemoglobin") AND (TEER OR "transcatheter edge-to-edge repair" OR "transcatheter mitral edge-to-edge repair" OR "edge-to-edge repair" OR MitraClip OR PASCAL OR "transcatheter mitral valve repair" OR "percutaneous mitral valve repair")	4
Embase	See below	See below
VHL	("mitral regurgitation" OR "mitral valve insufficiency" OR "mitral incompetence" OR "mitral valve incompetence" OR "mitral insufficiency") AND (anemia OR anaemia OR hemoglobin OR haemoglobin OR "low hemoglobin" OR "low haemoglobin") AND (TEER OR "transcatheter edge-to-edge repair" OR "transcatheter mitral edge-to-edge repair" OR "edge-to-edge repair" OR MitraClip OR PASCAL OR "transcatheter mitral valve repair" OR "percutaneous mitral valve repair")	38
Cochrane Library	("mitral regurgitation" OR "mitral valve insufficiency" OR "mitral incompetence" OR "mitral valve incompetence" OR "mitral insufficiency") AND (anemia OR anaemia OR hemoglobin OR haemoglobin OR "low hemoglobin" OR "low haemoglobin") AND (TEER OR "transcatheter edge-to-edge repair" OR "transcatheter mitral edge-to-edge repair" OR "edge-to-edge repair" OR MitraClip OR PASCAL OR "transcatheter mitral valve repair" OR "percutaneous mitral valve repair")	No relevant systematic review on baseline anemia and outcomes after mitral TEER was identified; retrieved records were mainly unrelated studies or duplicates of already identified literature.
clinicalTrials.gov	Condition/disease: Mitral Regurgitation Other terms: Anemia Intervention/treatment: MitraClip Implant	Retrieved records were mainly general MitraClip/mitral repair studies and were not specifically focused on baseline anemia as the exposure of interest.
Google Scholar	mitraclip anemia "preprocedural anemia" MitraClip "baseline anemia" MitraClip "impact of preprocedural anemia" MitraClip "transcatheter edge-to-edge repair" anemia. The first 6 pages were screened for each search query.	Retrieved records were mainly duplicates, conference abstracts, and studies not directly relevant to the review question. The key eligible studies had already been identified through the primary database searches. No relevant published systematic review or meta-analysis on the exact topic was identified.

**Table 2:** Stepwise Embase search strategy. TEER: transcatheter edge-to-edge mitral valve repair; MR: mitral regurgitation.

No.	Term	Result
1	MR (exp mitral valve regurgitation/)	64893
2	Anemia(anemia/)	544636
3	TEER (exp transcatheter edge to edge mitral valve repair/)	2123
4	TEER (or exp mitral valve clip/)	4008
5	3 or 4	5302
6	1 and 2 and 5	85

Eligibility criteria

Original primary observational studies assessing outcomes following mitral TEER in adult patients (≥18 years) with and without documented baseline preprocedural anemia were eligible for inclusion. We included English-language human studies that clearly defined anemia status prior to the procedure, compared anemic and non-anemic patients, provided sufficient data for this comparison, and reported at least one clinical outcome of interest. We excluded pediatric patients (<18 years), conference abstracts, review articles, meta-analyses, editorials, commentaries, letters lacking analyzable original data, case reports, case series, gray literature, non-human studies, and non-English publications. Studies evaluating surgical mitral repair or non-mitral structural procedures, studies not reporting extractable or analyzable associations between pre-procedural anemia and post-procedural outcomes, studies focused on post-procedural rather than pre-procedural anemia, and duplicate publications were also excluded.

Selection process

**Figure 1:**
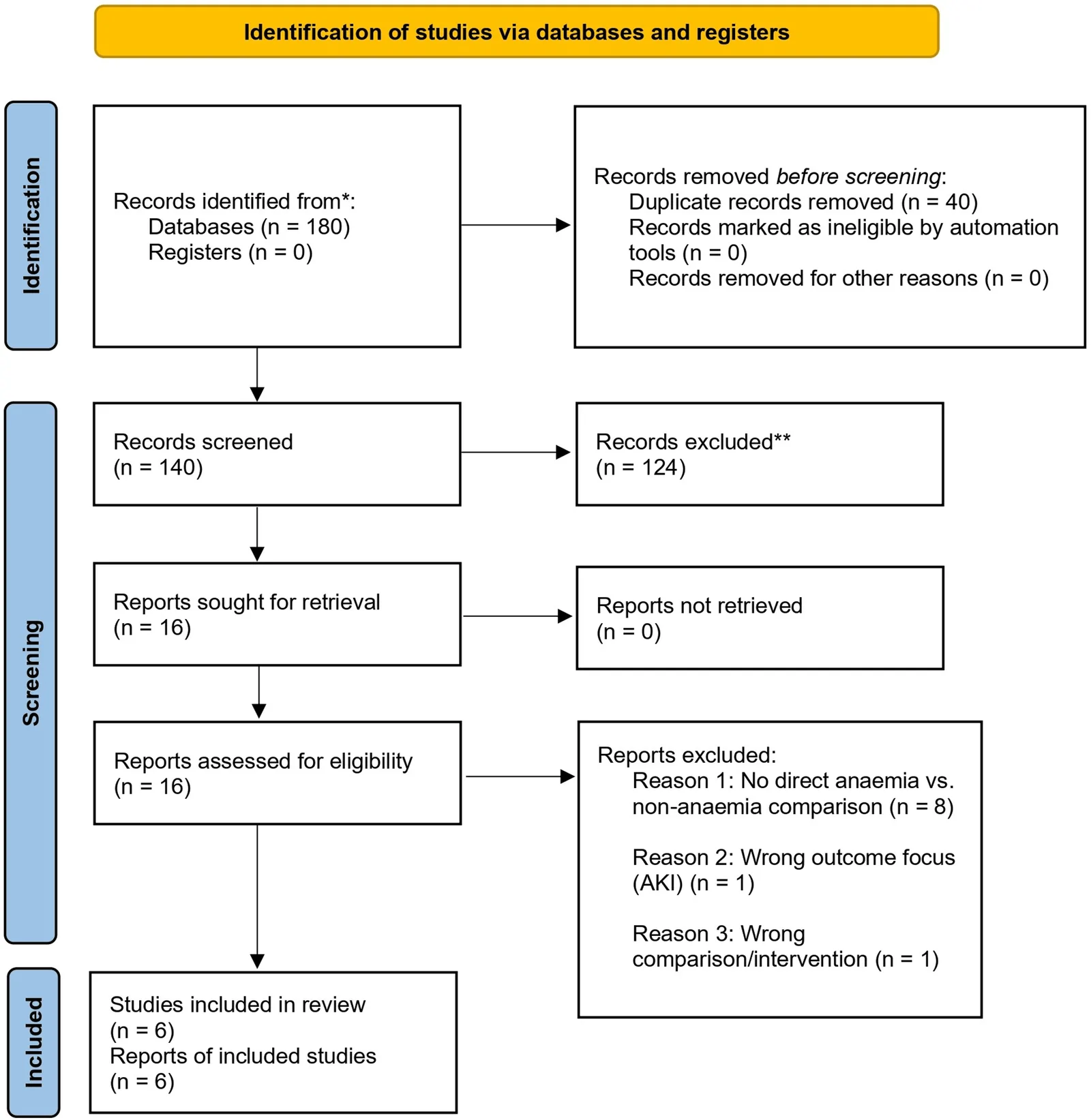
PRISMA 2020 flow diagram of study selection. AKI: acute kidney injury; PRISMA: Preferred Reporting Items for Systematic reviews and Meta-Analyses.

All identified records were imported into EndNote version 21.3 (Clarivate, Philadelphia, PA, USA), and duplicates were removed. Two reviewer pairs independently screened titles and abstracts and assessed full texts using the predefined eligibility criteria. Any disagreements were settled, with input from a senior reviewer when needed. The study selection process was summarized in a PRISMA 2020 flow diagram [15] (Figure 1).

Data collection process

The same two reviewer pairs independently extracted data using a standardized form. Extracted data were cross-checked for accuracy, with disagreements resolved by consensus.

Data items

As reported in Tables 3, 4, the following data were extracted from each included study, where available: study identification details, methodological and population characteristics, exposure-related variables, baseline clinical characteristics, and outcome data. Study quality was evaluated using the Newcastle-Ottawa Scale (NOS) [16]. Statistical analyses were performed using R software with the meta package [17].

**Table 3:** Study characteristics of included studies. NR: not reported.

First author	Year	Country	Study design	Study period	Follow-up period	Total	Anemia (n)	Non-anemia (n)
Iliadis et al. [18]	2020	Germany	Prospective	06/2016-10/2017	6 weeks; 14 ± 6 months	130	65	65
Khurrami et al. [19]	2023	Germany	Retrospective	09/2008-07/2019	5.5 years (max 12.3 years)	833	513	320
Kaneko et al. [20]	2018	Germany	Retrospective	03/2009-12/2016	569 ± 544 days	392	220	172
Bhardwaj et al. [21]	2020	USA	Retrospective	01/2011-09/2015	In-hospital	4382	978	3404
Al Yami et al. [22]	2024	USA	Retrospective	2016-2019	In-hospital; 30-,90-,180-day follow-up	28,995	1434	27,561
Hellhammer et al. [23]	2015	Germany	Prospective	NR	12 months	80	41	39

**Table 4:** Baseline patient characteristics of included studies. AF: atrial fibrillation; Adv. NYHA class: advanced New York Heart Association class; CABG: coronary artery bypass grafting; CAD: coronary artery disease; CKD: chronic kidney disease; COPD: chronic obstructive pulmonary disease; ESRD: end-stage renal disease; HTN: hypertension; LVEF: left ventricular ejection fraction; MI: myocardial infarction; NT-proBNP: N-terminal pro-B-type natriuretic peptide; PCI: percutaneous coronary intervention; PVD: peripheral vascular disease; NR: not reported. Continuous variables are presented as mean ± standard deviation (SD) or median (IQR), as reported by the original studies.

First author	Age	Male sex	Female sex	Adv. NYHA class	HTN	LVEF	CKD	Diabetes	AF	CAD	Prior MI	Prior PCI	Prior CABG	COPD/pulmonary disease	EuroScore	NT-proBNP/BNP	ESRD	PVD
Iliadis et al. [18]	80 (8)	66 (50.8%)	64 (49.2%)	125 (96.2%)	97 (74.6%)	71/129 (55.0%)	97 (74.6%)	27 (20.8%)	90 (69.2%)	79 (60.8%)	29 (22.3%)	NR	NR	21/129 (16.3%)	38 (29.2%)	2108 (2725)	NR	18 (13.8%)
Khurrami et al. [19]	77.1 (71.5, 82.1)	494 (59.3%)	339 (40.7%)	769 (93.8%)	73.2%	40.7 (28.7, 53.3)	578 (71.3%)	229 (27.8%)	565 (68.3%)	549 (66.2%)	227 (27.5%)	353 (42.9%)	197 (23.9%)	167 (20.3%)	19.7% (11.5-31.8)	4010 (1875, 8268)	NR	98 (12.1%)
Kaneko et al. [20]	NR	263 (67.1%)	129 (32.9%)	106 (28.7%)	147 (37.5%)	NR	243 (74.1%)	238 (60.7%)	236 (60.2%)	NR	96 (24.5%)	164 (41.8%)	88 (22.5%)	85 (21.7%)	NR	NR	NR	NR
Bhardwaj et al. [21]	77 (67–84)	2494 (56.9%)	1888 (43.1%)	NR	3138 (71.6%)	NR	1507 (34.4%)	1100 (25.1%)	2524 (57.6%)	NR	627 (14.3%)	666 (15.2%)	1060 (24.2%)	1161 (26.5%)	NR	NR	145 (3.3%)	495 (11.3%)
Al Yami et al. [22]	NR	15,709 (54.2%)	13,289 (45.8%)	NR	20,324 (70.1%)	NR	NR	NR	NR	NR	4754 (16.4%)	5160 (17.8%)	6104 (21.1%)	6290 (21.7%)	NR	NR	NR	NR
Hellhammer et al. [23]	73 ± 10.9	54 (67.5%)	26 (32.5%)	73 (91.3%)	NR	42.1 ± 14.9	NR	34 (42.5%)	48 (60%)	50 (62.5%)	NR	6 (7.5%)	24 (30.0%)	17 (21.3%)	20.0 ± 17.3	4482.1	2 (4.9%)	NR

The prespecified primary outcome was all-cause mortality. Prespecified secondary outcomes included cardiac morbidity and cardiovascular events, readmission, acute kidney injury, bleeding-related outcomes, length of stay (LOS), functional or quality-of-life outcomes where reported, and procedure-related complications. Additional clinically relevant outcomes reported by the included studies, including myocardial infarction, stroke, cardiogenic shock, pericardial complications, respiratory failure, venous thromboembolism (VTE), infection, gastrointestinal complications, composite outcomes, and discharge-related outcomes, were also extracted to provide a comprehensive assessment of the available evidence.

Across the included studies, anemia was generally defined according to World Health Organization hemoglobin thresholds, typically hemoglobin <13 g/dL in men and <12 g/dL in women. Where studies used administrative diagnostic coding rather than directly reported hemoglobin thresholds, the study-specific definition was retained.

When multiple effect estimates were reported for the same outcome, adjusted estimates were preferentially extracted. Where more than one adjusted model was available, the estimate from the model with the most comprehensive multivariable adjustment was selected. The covariates included in these models varied across studies and comprised combinations of demographic characteristics, cardiovascular disease severity, renal function, and relevant comorbidities.

Study-reported restricted multivariable models were used only when the relevant outcome was unavailable in the primary model. If required, effect estimates were transformed to the log scale, and the corresponding standard errors were derived from the reported confidence intervals.

Study quality assessment

Two reviewers independently assessed study quality using the Newcastle-Ottawa Scale (NOS) for cohort studies [16], with disagreements resolved by consensus. Studies were classified as good quality (7-9 points), fair quality (3-6 points), or poor quality (0-2 points).

Effect measures

Hazard ratios (HRs) and adjusted odds ratios (aORs), along with 95% confidence intervals (CIs), were used as effect measures according to outcome type and reporting format. HRs were used for time-to-event outcomes, whereas ORs with 95% CIs were used for dichotomous in-hospital outcomes.

Synthesis methods

Outcomes were synthesized according to their clinical relevance and the comparability of outcome definitions, time points, and effect measures. Quantitative meta-analysis was performed only when at least two studies reported sufficiently comparable data for the same outcome; otherwise, the available evidence was summarized narratively.

Pooled analyses used a random-effects model with restricted maximum likelihood (REML) estimation of between-study variance (τ²) and Hartung-Knapp adjustment. Statistical heterogeneity was assessed using Cochran's Q, I², and τ² statistics. Subgroup analyses and meta-regression were not performed because the small number of studies contributing to each meta-analysis precluded meaningful exploration of potential sources of heterogeneity. Prediction intervals were not reported because of the very small number of studies contributing to individual meta-analyses, which would result in highly uncertain and potentially uninformative estimates. A sensitivity analysis was performed for long-term mortality by including Iliadis et al. [18] in addition to the primary analysis studies.

Formal assessment of publication bias was not performed because the small number of studies contributing to each meta-analysis precluded reliable evaluation of funnel-plot asymmetry or statistical tests for small-study effects.

Results

Study Selection

The study selection process is illustrated in Figure 1. Of 180 records identified, 40 duplicates were removed, and 140 records were screened. Sixteen reports underwent full-text assessment; 10 were excluded, leaving six studies for inclusion in the systematic review and meta-analysis.

Study Characteristics

The characteristics of the included studies are detailed in Table 3. Six observational studies were included. Collectively, these studies included 34,812 patients, of whom 3,251 had baseline anemia and 31,561 did not. The sample sizes varied from 80 to 28,995 patients. The follow-up periods ranged from in-hospital outcomes only to long-term follow-up, with the longest reported follow-up reaching 12.3 years.

Study Quality Assessment

All included studies were of good quality, with NOS scores ranging from 7 to 9 (Table 5).

**Table 5:** Quality assessment of included studies.

Study		Newcastle-Ottawa quality assessment items								Total score	Quality
		Selection				Comparability	Outcome				
Author	Year	Representativeness of exposed cohort	Selection of nonexposed cohort	Ascertainment of exposure	Outcome does not present at the start		Assessment of outcome	Sufficient follow-up duration	Adequate follow-up		
Iliadis et al. [18]	2020	1	1	1	1	2	1	1	1	9	Good
Khurrami et al. [19]	2023	1	1	1	1	2	1	1	0	8	Good
Kaneko et al. [20]	2018	1	1	1	1	2	1	1	0	8	Good
Bhardwaj et al. [21]	2020	1	1	1	1	2	1	1	1	9	Good
Al Yami et al. [22]	2024	1	1	1	1	2	1	1	1	9	Good
Hellhammer et al. [23]	2015	1	1	1	1	0	1	1	1	7	Good

Quantitative Synthesis

**Figure 2:**
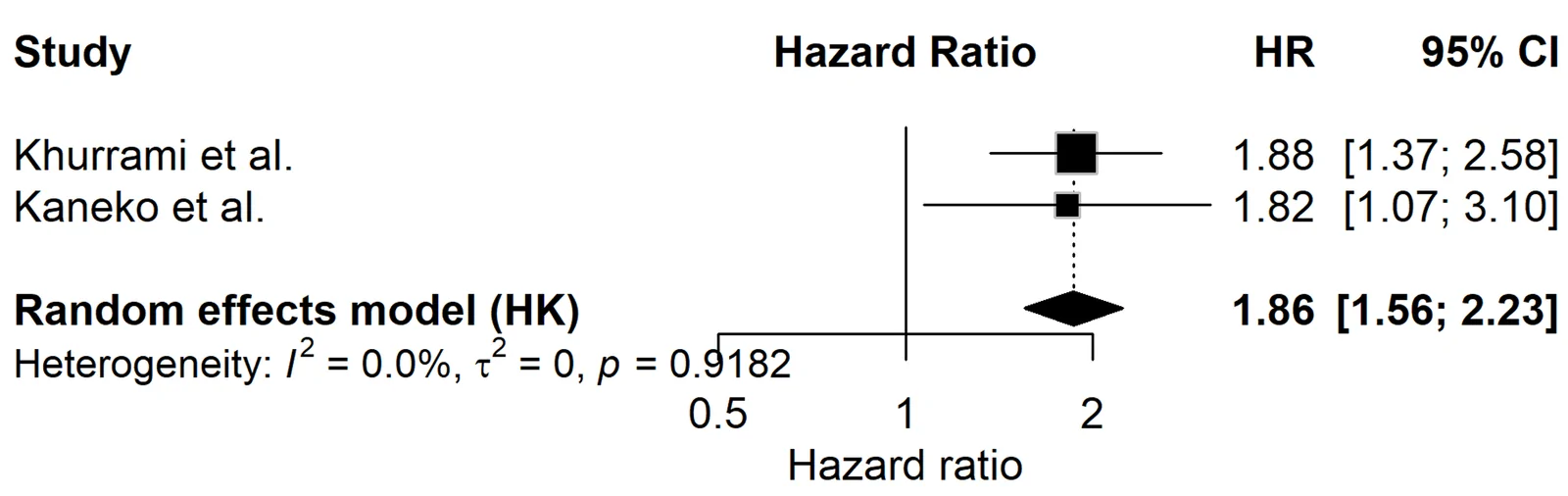
Forest plot of hazard ratios for long-term mortality associated with baseline anemia after mitral TEER. Khurrami et al. [19] and Kaneko et al. [20]. HR: hazard ratio; CI: confidence interval; HK: Hartung-Knapp method; TEER: transcatheter edge-to-edge mitral valve repair.

Mortality outcomes: For long-term mortality, pooling data from Khurrami et al. [19] and Kaneko et al. [20] showed that baseline anemia was associated with a significantly increased long-term mortality after TEER, with a pooled HR of 1.86 (95% CI 1.56-2.23; p = 0.014). There was no evidence of important heterogeneity (Q (1) = 0.01, p = 0.918; I² = 0%; τ² = 0). The findings are shown in Figure 2.

**Figure 3:**
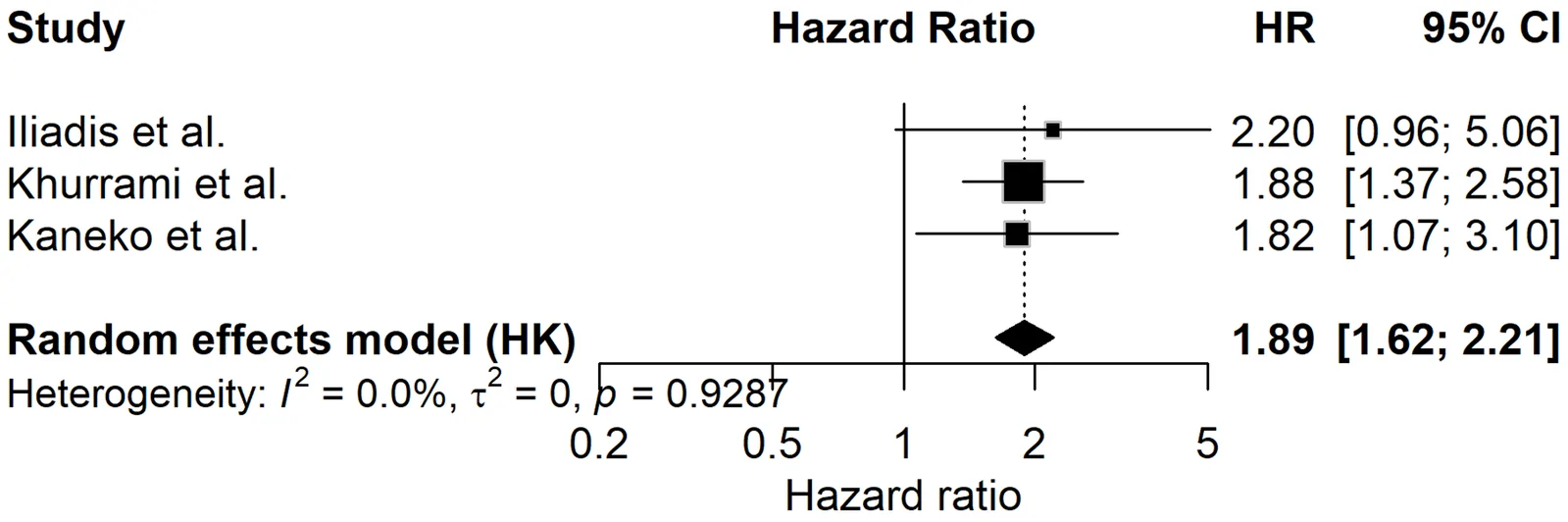
Forest plot of the sensitivity analysis of long-term mortality after TEER. Studies included: Iliadis et al. [18], Khurrami et al. [19], and Kaneko et al. [20]. HR: hazard ratio; CI: confidence interval; HK: Hartung-Knapp method; TEER: transcatheter edge-to-edge mitral valve repair.

Sensitivity analysis, including Iliadis et al. [18] for which adjustment reporting was less explicit, yielded findings consistent with the primary analysis. The pooled result remained statistically significant. When Khurrami et al. [19], Kaneko et al. [20], and Iliadis et al. [18] were pooled together, baseline anemia remained associated with increased long-term mortality after TEER, with a pooled HR of 1.89 (95% CI 1.62-2.21; p = 0.003). Heterogeneity remained negligible (Q (2) = 0.15, p = 0.928; I² = 0%; τ² = 0). The sensitivity analysis is illustrated in Figure 3.

**Figure 4:**
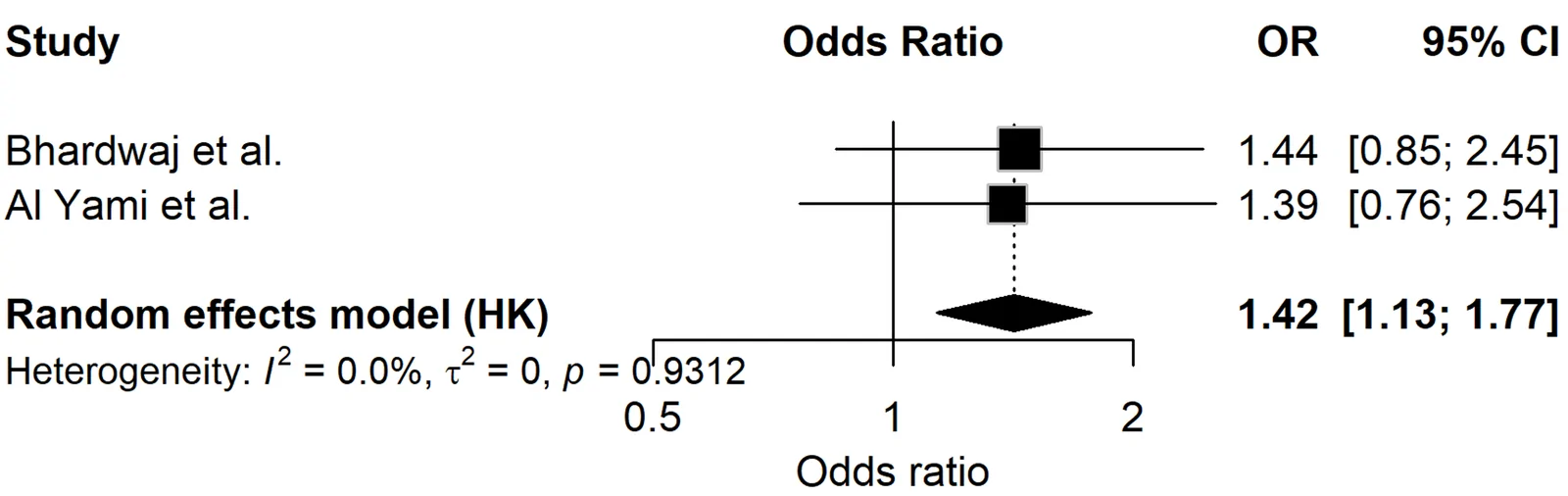
Forest plot of in-hospital mortality reported as adjusted odds ratios according to baseline anemia status after mitral TEER. Bhardwaj et al. [21] and Al Yami et al. [22]. OR: odds ratio; CI: confidence interval; HK: Hartung-Knapp method; TEER: transcatheter edge-to-edge mitral valve repair.

For in-hospital mortality, a pooled analysis of Bhardwaj et al. [21] and Al Yami et al. [22] showed higher odds in anemic patients, with a pooled aOR of 1.42 (95% CI 1.13-1.77; p = 0.032). No important heterogeneity was observed (Q (1) = 0.01, p = 0.931; I² = 0%; τ² = 0). The corresponding forest plot is shown in Figure 4.

Procedural Complications

**Figure 5:**
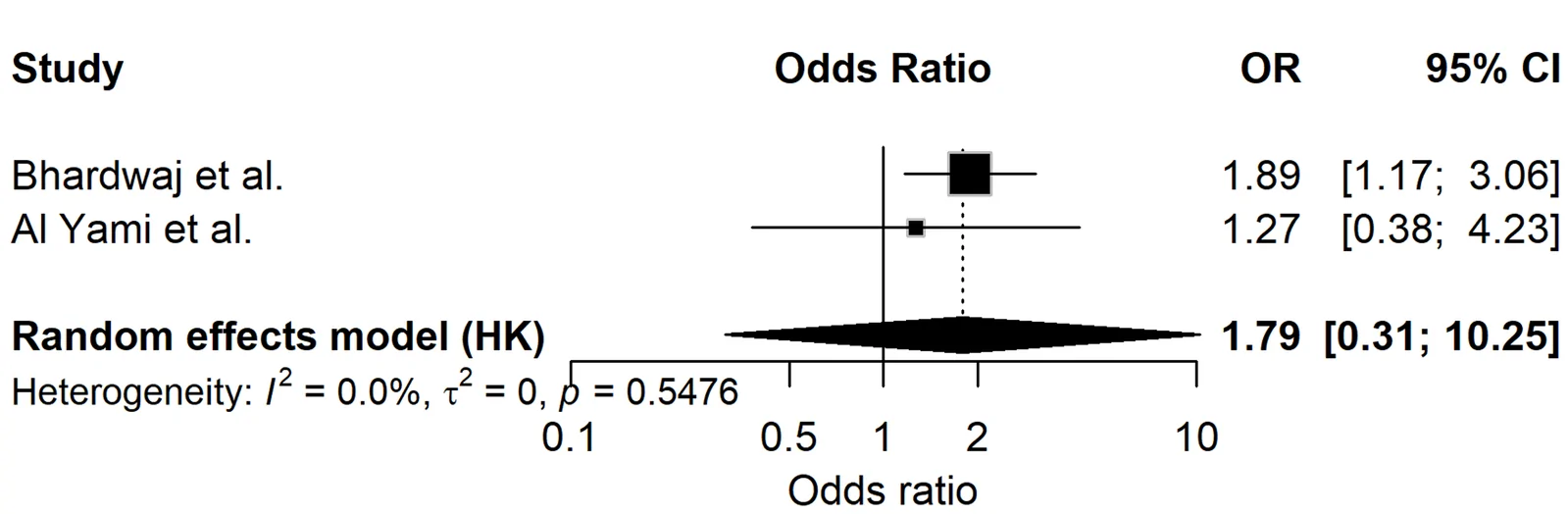
Forest plot of odds ratios for pericardial complications associated with baseline anemia after mitral TEER. Bhardwaj et al. [21] and Al Yami et al. [22]. OR: odds ratio; CI: confidence interval; HK: Hartung-Knapp method; TEER: transcatheter edge-to-edge mitral valve repair.

Pooled analysis of pericardial complications from Bhardwaj et al. [21] and Al Yami et al. [22] did not demonstrate a statistically significant association with baseline anemia (pooled aOR 1.79, 95% CI 0.31-10.25; p = 0.148). The wide confidence interval indicates substantial imprecision in the pooled estimate. Observed statistical heterogeneity was low (Q(1) = 0.36, p = 0.548; I² = 0%; τ² = 0), although heterogeneity estimates are inherently uncertain with only two contributing studies. The findings are shown in Figure 5.

Narrative Synthesis of Non-pooled Outcomes

Several clinically relevant outcomes could not be quantitatively pooled because of heterogeneity in outcome definitions, follow-up periods, reporting formats, and/or the availability of comparable adjusted effect estimates. These outcomes were therefore synthesized narratively, with the principal findings summarized below and comprehensively presented in Table 6.

**Table 6:** Summary of outcomes and synthesis approaches across included studies. *Iliadis et al. [18] was included only in the sensitivity analysis for long-term mortality. HR: hazard ratio; aOR: adjusted odds ratio; CI: confidence interval; REML: restricted maximum likelihood; AKI: acute kidney injury.

Outcome	Studies contributing to synthesis	Effect measure/statistical model (no. of studies)	Main findings	Synthesis approach
Long-term mortality	Iliadis et al. [18]*; Khurrami et al. [19]; Kaneko et al. [20]	HR; random-effects REML with Hartung-Knapp adjustment (two primary; three sensitivity)	Primary pooled HR 1.86 (95% CI 1.56-2.23); sensitivity pooled HR 1.89 (95% CI 1.62-2.21).	Quantitative + sensitivity
In-hospital mortality	Bhardwaj et al. [21]; Al Yami et al. [22]	aOR; random-effects REML with Hartung-Knapp adjustment (two studies)	Pooled aOR 1.42 (95% CI 1.13-1.77).	Quantitative
Pericardial complications	Bhardwaj et al. [21]; Al Yami et al. [22]	aOR; random-effects REML with Hartung-Knapp adjustment (two studies)	Pooled aOR 1.79 (95% CI 0.31-10.25).	Quantitative
Acute kidney injury	Bhardwaj et al. [21]; Al Yami et al. [22]; Hellhammer et al. [23]	-	Increased AKI risk was reported in anemic patients, although findings varied according to AKI definition and severity.	Narrative
Long-term composite outcome	Iliadis et al. [18]; Khurrami et al. [19]; Al Yami et al. [22]; Hellhammer et al. [23]	-	Iliadis et al. and Khurrami et al. reported increased adjusted risk of death/mortality and heart failure hospitalization in anemic patients; other composite outcomes were not sufficiently comparable for pooling.	Narrative
Heart failure-related outcomes	Iliadis et al. [18]; Al Yami et al. [22]	-	Generally higher risk in anemic patients; narratively synthesized because of differences in outcome definition and timing.	Narrative
Myocardial infarction	Al Yami et al. [22]	-	Higher adjusted odds of myocardial infarction reported in anemic patients.	Narrative
Blood transfusion/bleeding-related outcomes	Iliadis et al. [18]; Bhardwaj et al. [21]; Hellhammer et al. [23]	-	Transfusion or bleeding-related events were generally more frequent in anemic patients overall.	Narrative
Stroke	Bhardwaj et al. [21]; Al Yami et al. [22]; Hellhammer et al. [23]	-	No consistent statistically significant association across studies.	Narrative
Length of stay	Iliadis et al. [18]; Bhardwaj et al. [21]; Al Yami et al. [22]	-	Longer hospitalization in anemic patients.	Narrative
Cardiogenic shock	Bhardwaj et al. [21]; Al Yami et al. [22]	-	No significant association overall.	Narrative
Respiratory outcomes	Bhardwaj et al. [21]; Hellhammer et al. [23]	-	No significant association overall.	Narrative
Venous thromboembolism	Bhardwaj et al. [21]	-	No significant association overall.	Narrative
Infection	Bhardwaj et al. [21]; Hellhammer et al. [23]	-	No significant association overall.	Narrative
Gastrointestinal complications	Bhardwaj et al. [21]	-	No significant association overall.	Narrative
Discharge/readmission-related outcomes	Bhardwaj et al. [21]; Al Yami et al. [22]	-	Lower odds of routine discharge and higher readmission burden in anemic patients.	Narrative

Cardiovascular and Renal Outcomes

Long-term composite outcomes were reported by two studies. Iliadis et al. [18] found that anemia was independently associated with an increased risk of the combined endpoint of mortality and heart failure hospitalization (adjusted HR 2.29; 95% CI 1.08-4.85; p = 0.030). Similarly, Khurrami et al. [19] reported an increased adjusted risk of the composite endpoint of death or heart failure rehospitalization among patients with anemia (adjusted HR 1.39; 95% CI 1.08-1.79; p = 0.009). Although the composite endpoints were clinically comparable and both studies demonstrated associations in the same direction, quantitative pooling was not retained because only two studies were available and the resulting random-effects estimate was highly imprecise. Hellhammer et al. [23] and Al Yami et al. [22] reported in-hospital major adverse cardiovascular and cerebrovascular event (MACCE) outcomes, which were summarized narratively because they were not equivalent to the long-term composite outcome of death and heart failure hospitalization and were not sufficiently comparable for pooling.

For renal outcomes, Al Yami et al. [22] reported that baseline anemia was independently associated with increased odds of in-hospital AKI (aOR 2.21, 95% CI 1.81-2.60). Bhardwaj et al. [21] separately evaluated AKI without dialysis and AKI requiring dialysis; anemia was associated with increased adjusted odds of both AKI without dialysis (aOR 1.53, 95% CI 1.21-1.93) and AKI requiring dialysis (aOR 3.24, 95% CI 1.75-5.99). In contrast, Hellhammer et al. [23] specifically reported stage III AKI, which occurred in 4.9% of patients with anemia and 5.1% of those without anemia (p = 0.959), and provided no adjusted effect estimate. Given the differences in renal outcome reporting and the limited number of studies providing comparable adjusted estimates, these findings were not quantitatively pooled.

For heart failure-related outcomes, Iliadis et al. [18] reported that anemia was associated with a higher risk of heart failure hospitalization during follow-up (HR 3.28, 95% CI 1.06-10.19; p = 0.040). Al Yami et al. [22] also reported significantly higher odds of in-hospital heart failure among anemic patients (aOR 1.75, 95% CI 1.28-2.30; p < 0.001).

For myocardial infarction, Al Yami et al. [22] reported higher adjusted odds of myocardial infarction among anemic patients undergoing TEER (aOR 1.54, 95% CI 1.01-2.33; p = 0.041).

For cardiogenic shock, Bhardwaj et al. [21] reported numerically higher odds of in-hospital cardiogenic shock in anemic patients (aOR 1.37, 95% CI 0.97-1.92; p = 0.074). Al Yami et al. similarly found no significant difference in in-hospital cardiogenic shock between anemic and non-anemic patients (p = 0.354) in the propensity-matched analysis.

For stroke, Al Yami et al. [22] reported no significant association between anemia and in-hospital stroke (aOR 1.85, 95% CI 0.75-4.54; p = 0.177). Hellhammer et al. [23] reported no significant difference in perioperative stroke between anemic and non-anemic patients (1/41 (2.4%) vs 0/39 (0.0%); p = 0.326). Bhardwaj et al. [21] also reported perioperative stroke, but no directly usable effect estimate was available for quantitative pooling.

Other Clinical and Procedural Outcomes

For blood transfusion and bleeding-related outcomes, Bhardwaj et al. [21] reported increased odds of in-hospital blood transfusion among anemic patients (aOR 1.33, 95% CI 1.09-1.62; p < 0.001). Iliadis et al. [18] also reported more frequent blood transfusion after MitraClip in anemic patients but did not provide a directly usable adjusted effect estimate. Hellhammer et al. [23] reported no significant difference in postoperative transfusion requirements between groups (39.0% vs 28.2%; p = 0.269), and provided no directly usable adjusted effect estimate for pooling.

For length of stay (LOS), Bhardwaj et al. [21] reported that anemia was associated with prolonged LOS (β = 1.17, adjusted p < 0.001). Iliadis et al. [18] stated that hospitalization was significantly prolonged in anemic patients, although no directly usable adjusted effect estimate was provided. Al Yami et al. also reported that LOS was longer in the anemic group and presented descriptive readmission LOS data, but these were not directly extractable for pooled meta-analysis.

For respiratory outcomes, Bhardwaj et al. [21] reported in-hospital postoperative respiratory failure and found no significant association with anemia (aOR 0.85, 95% CI 0.56-1.30; p = 0.457). Hellhammer et al. [23] reported ventilation >24 hours and similarly found no significant difference between anemic and non-anemic patients (p = 0.603).

Only one study reported venous thromboembolism data. Bhardwaj et al. [21] found no significant association between anemia and VTE (aOR 1.32, 95% CI 0.67-2.57; p = 0.418)

For infection-related outcomes, Bhardwaj et al. [21] reported postoperative infection and found no statistically significant association with anemia (aOR 1.25, 95% CI 0.61-2.55; p = 0.543). Hellhammer et al. [23] also reported no statistically significant difference in sepsis between anemic and non-anemic patients (p = 0.586).

Limited comparative data were available for gastrointestinal complications. Bhardwaj et al. [21] reported no significant association between anemia and gastrointestinal complications (aOR 0.73, 95% CI 0.45-1.19; p = 0.205)

For discharge-related outcomes and readmission, Bhardwaj et al. [21] reported that anemia was associated with lower odds of routine discharge (aOR 0.57, 95% CI 0.48-0.67; p < 0.001). Al Yami et al. [22] additionally evaluated 30-, 90-, and 180-day readmission outcomes and reported an overall higher readmission burden in the anemic group, but these data were not sufficiently comparable for pooled analysis.

Discussion

To our knowledge, this is the first full systematic review and meta-analysis specifically evaluating the association between baseline preprocedural anemia and clinical outcomes after mitral TEER. Several additional TEER studies evaluating anemia or hemoglobin as prognostic variables were identified during the literature search but did not meet the predefined eligibility criteria for inclusion in the systematic review because they did not provide a direct anemia-versus-non-anemia comparison. The principal findings of this review can be summarized as follows. First, patients with baseline anemia experienced higher mortality after TEER, including both in-hospital and long-term mortality. Second, the pooled analysis of pericardial complications did not demonstrate a statistically significant association. Third, outcomes not amenable to quantitative pooling were synthesized narratively; these generally suggested less favorable outcomes among patients with anemia, including renal complications, long-term composite outcomes of mortality and heart failure hospitalization, heart failure-related events, myocardial infarction, blood transfusion, and length of stay. Collectively, these findings support the prognostic relevance of baseline anemia in patients undergoing TEER, while the evidence for individual non-mortality outcomes remains limited and should be interpreted cautiously.

Our findings are consistent with prior observational TEER literature showing that low hemoglobin or anemia has prognostic importance after mitral TEER. Bansal et al. [24] showed that lower baseline hemoglobin was independently associated with higher 30-day out-of-hospital mortality after mitral TEER. Buccheri et al. [14] identified low hemoglobin or anemia as independent predictors of one-year mortality after MitraClip therapy. Over a longer follow-up period, Raposeiras-Roubín et al. [25] also identified anemia as an independent predictor of mortality during follow-up in patients undergoing TEER. In addition, Esposito et al. [26] showed that lower hemoglobin independently predicted cardiac mortality, while Kaya et al. [27] reported that lower hemoglobin remained independently associated with mortality after multivariable adjustment. Beyond mortality alone, Scotti et al. [28] incorporated hemoglobin into the CITE score for prediction of two-year death or heart failure hospitalization. Taken together, these studies support the broader prognostic relevance of baseline hematologic status and align with the pooled mortality findings of the present meta-analysis.

Although mortality was the most consistent endpoint, the effect of anemia likely extends beyond that. Prior TEER literature also suggests associations between baseline anemia and additional postprocedural outcomes. Armijo et al. [29] found that baseline anemia was an independent predictor of acute kidney injury after percutaneous TEER, and that postprocedural AKI was associated with substantially worse downstream outcomes. Similar signals were observed across several studies included in the present review [21-23], although the reported renal endpoints were not directly comparable. Collectively, these findings suggest a possible association between baseline anemia and increased renal complications following TEER; however, the heterogeneity of outcome definitions and limited comparative evidence preclude firm conclusions. Similarly, Lima et al. [30] also incorporated anemia into prediction models for 30-day readmission after TEER, supporting a broader association with an adverse early post-procedural course.

Several mechanisms may underlie this association. In TEER candidates, anemia may reflect a broader burden of illness, including advanced heart failure, renal dysfunction, inflammation, frailty, and reduced physiological reserve, all of which may contribute to reduced procedural tolerance and worse outcomes. In addition, reduced oxygen-carrying capacity may increase susceptibility to tissue hypoxia and organ dysfunction. In particular, preprocedural anemia has been associated with AKI following TEER, with proposed mechanisms including renal hypoxia, inflammatory responses, and oxidative stress. The prognostic relevance of anemia is also supported by prior TEER studies, including Armijo et al. [29], Khurrami et al. [19], and Kaneko et al. [20], in which anemia or lower hemoglobin consistently emerged as independent prognostic markers.

Clinical implications

These findings have practical implications for TEER assessment. Baseline anemia may represent a useful component of preprocedural assessment and risk stratification. This may help inform risk discussions with patients, support individualized procedural decision-making, and identify patients who may warrant closer postprocedural monitoring. Importantly, anemia may not only serve as a risk indicator but also as a potentially modifiable condition in certain patients. This raises an important clinical and research question as to whether preprocedural optimization of anemia could improve TEER outcomes. However, specific evidence-based recommendations for anemia correction before mitral TEER remain lacking. Although current evidence does not yet prove that correcting anemia before TEER improves outcomes, our findings suggest that this is an important question that warrants further evaluation in prospective studies.

Strengths and limitations

Key strengths of this review include its focused clinically relevant question, specifically synthesizing anemia versus non-anemia evidence in contrast to much of the prior TEER literature, which evaluated hemoglobin primarily as a continuous prognostic variable or as a component of broader risk scores. In addition, this review included a large overall study population, enhancing the clinical relevance of the findings. The inclusion of both quantitative and narrative synthesis allowed us to capture a wider range of clinically meaningful outcomes while maintaining methodological restraint when outcomes were too heterogeneous in definition, timing, or reporting to justify pooling. A further strength is the use of adjusted effect estimates where available.

This review also has important limitations. First, because all included studies were observational, causal relationships cannot be established, and despite preferential use of adjusted estimates and generally good NOS ratings, the covariates included in multivariable models varied across studies, and residual confounding may therefore remain. Second, some pooled analyses were based on only a small number of studies, which limited precision and contributed to wide confidence intervals for certain outcomes. Third, not all clinically relevant outcomes were supported by directly comparable adjusted effect estimates, and several endpoints therefore required narrative rather than quantitative synthesis. Fourth, anemia in this population is likely heterogeneous in etiology, potentially reflecting iron deficiency, chronic inflammation, renal dysfunction, or advanced heart failure, but these mechanisms were not consistently characterized across the included studies. In addition, the included studies did not permit reliable assessment of outcomes according to anemia severity or specific hemoglobin levels, limiting interpretation of whether particular hemoglobin thresholds carry differential prognostic significance in patients undergoing mitral TEER. Additionally, the exclusion of non-English publications and gray literature may introduce language and publication bias, potentially limiting the generalizability of our findings and resulting in underrepresentation of relevant evidence from other populations or settings.

## Conclusions

In conclusion, baseline preprocedural anemia was associated with worse clinical outcomes after mitral TEER, most consistently for mortality, and may also be associated with a broader pattern of adverse clinical outcomes. These findings suggest that anemia should be considered during preprocedural assessment. Further prospective studies are needed to determine whether targeted identification and optimization of anemia before the procedure can translate into improved post-TEER outcomes.
